# High-Resolution Imaging in Macular Telangiectasia Type 2: Case Series and Literature Review

**DOI:** 10.3390/diagnostics14131351

**Published:** 2024-06-25

**Authors:** Andrada Elena Mirescu, Florian Balta, Ramona Barac, Dan George Deleanu, Ioana Teodora Tofolean, George Balta, Razvan Cojanu, Sanda Jurja

**Affiliations:** 1 Department of Ophthalmology, “Ovidius” University of Medicine, 900470 Constanța, Romania; 2 Department of Ophthalmology, “Carol Davila” University of Medicine and Pharmacy, 050747 Bucharest, Romania; 3 Academy of Romanian Scientists, 030167 Bucharest, Romania; 4 Retina Clinic, 014142 Bucharest, Romania; 5 Clinical Emergency Eye Hospital, 030167 Bucharest, Romania; 6 Department of Ophthalmology, University Emergency Hospital, 050098 Bucharest, Romania; 7 Department of Biophysics, Faculty of Medicine, “Carol Davila” University of Medicine and Pharmacy, 050747 Bucharest, Romania; 8 Department of Ophthalmology, County Clinical Emergency Hospital of Constanta, 900591 Constanta, Romania

**Keywords:** macular teleangiectasia, high resolution imaging of the retina

## Abstract

Background: Macular telangiectasia (MacTel), also known as idiopathic juxtafoveolar telangiectasis (IJFTs), involves telangiectatic changes in the macular capillary network. The most common variant, MacTel type 2, has distinct clinical features and management strategies. Methods: This study offers a comprehensive review of MacTel and focuses on a series of three patients diagnosed with MacTel type 2 in our clinic. A meticulous ophthalmological evaluation, augmented by high-resolution imaging techniques like optical coherence tomography (OCT), OCT angiography (OCT-A), fundus autofluorescence (FAF), fluorescein angiography (FA), and adaptive optics (AOs) imaging, was conducted. Results: The findings revealed normal anterior segment features and a grayish discoloration in the temporal perifoveal area on fundus examination. OCT exhibited hyporeflective cavities in the inner and outer neurosensory retina, along with other changes, while OCT-A identified retinal telangiectatic vessels in the deep capillary plexus. FAF demonstrated increased foveal autofluorescence, while FA initially detected telangiectatic capillaries followed by diffuse perilesional leakage in the later phase. Adaptive optics images showed the cone mosaic pattern. Notably, one patient developed a macular hole as a complication, which was successfully managed surgically. Conclusion: This study underscores the challenges in diagnosing and managing MacTel, emphasizing the importance of a multidisciplinary approach and regular follow-ups for optimal outcomes.

## 1. Introduction

Macular telangiectasia (MacTel), alternatively referred to as “idiopathic juxtafoveolar telangiectasis” (IJFT), denotes a diverse range of clinical conditions marked by telangiectatic changes in the macular capillary network of one or both eyes. These conditions vary in appearance, are presumed to be pathogenesis, and necessitate different management approaches [1]. These entities are distinguished from the generalized retinal telangiectasis of the “idiopathic peripheral retinal telangiectasia” (IPT), also known as Coats disease [2]. They are additionally set apart from the more common retinal telangiectasis, which arises as a secondary effect of inflammatory or vascular conditions such as retinal vein occlusion, diabetes, irradiation, or the obstruction of the carotid artery [1,3].

The term “idiopathic juxtafoveolar telangiectasis” was first used by Gass and Oyakawa in 1982 to describe the clinical findings of twenty-seven adult patients with a decrease in visual acuity (VA) in one or both eyes (BE), along with other complaints, for example, blurred vision, metamorphopsia, and a positive scotoma. They also introduced the first classification of these entities into four groups based predominantly on biomicroscopical and fluorescein angiographic (FA) features [4]. The classification was further revised in 1993 by Gass and Blodi, subdividing IJFT into three distinct groups—1, 2, and 3—with additional subgroups in each (A and B) [5].

In 2006, Yannuzzi et al. proposed a streamlined classification system for IJFT, drawing upon novel clinical insights and observations from optical coherence tomography (OCT) and OCT angiography (OCTA) imaging. Nonetheless, their revision concurs with the already established Gass–Blodi model. They proposed the term “idiopathic macular telangiectasia” (IMT) with the following two distinct types: Type 1 (or “aneurysmal telangiectasia”), which is the second most common form of IJFT; and Type 2 (or “perifoveal telangiectasia”), equivalent to IJFT group 2A, which is the most common type of IJFT [1,6].

The remaining group, 2B, described by Gass and Blodi, was omitted from Yannuzzi’s classification because of its rarity [1]. The third group of the IJFT classification (3A and 3B), also known as “occlusive telangiectasia”, was omitted due to its rarity and the presence of perifoveal capillary nonperfusion, secondary to an ocular manifestation of familial systemic or cerebral disease, as the primary abnormality, rather than macular telangiectasia [6]. Yannuzzi et al. also streamlined the five stages of group 2A, initially proposed by Gass and Blodi, into two distinct stages with therapeutic and prognostic implications as follows: nonproliferative and proliferative stages [6].

### 1.1. Type 1—Idiopathic Macular Telangiectasia Type 1 or “Aneurysmal Telangiectasia”

Idiopathic macular telangiectasia Type 1 predominantly affects males and typically manifests unilaterally in about 97% of cases. While symptoms can emerge at any age, the average age at presentation is around 40 years [1].

The hallmark clinical features include prominent telangiectatic retinal capillaries with aneurysmal dilations accompanied by macular edema and lipid deposits. Typically, telangiectasis affects the temporal side of the fovea, namely an area equivalent to two-disc diameters. Macular edema and exudation stand as the primary culprits behind visual impairment in these patients. Visual acuity loss can vary, with a median acuity of 20/40 noted at presentation in Gass’s series. Nevertheless, certain patients may sustain exceptional visual acuity for years without intervention, only to experience a deterioration to a pathological state later in life. Spontaneous resolution is observed in some cases [1].

In addition to the core clinical manifestations mentioned earlier, patients with IMT may also exhibit focal vascular alterations extending outward from the central affected area [1]. Capillary, venular, and arteriolar aneurysms represent the primary features of Coats’ disease. Yannuzzi et al. proposed that IMA Type 1 is essentially a variant of Coats’ disease, uniquely localized to the macular region [2,6]. Peripheral aneurysmal telangiectasias typically exhibit a larger extent compared to Coats’ disease in the macular region and are more frequently associated with areas of hypoperfusion [2].

It is important to highlight that Gass and Blodi distinguished between two subgroups within IJFT 1: Group 1A, as described previously, and Group 1B, which shares identical clinical features but is limited to an area of two clock hours or less, often exhibiting superior VA [1,5]. Since these limited changes have a tendency to progress into a more extensive disease over time, Yannuzzi et al. proposed integrating the two groups into a unified classification system [6].

In cases where progressive visual loss occurs, treatment with laser photocoagulation appears to be effective in inducing a resolution in exudation and improving or stabilizing visual acuity in carefully selected patients [1]. Photocoagulation was initially recommended by Gass and Oyakawa in 1982 and continues to be a cornerstone of treatment strategies to this day [4]. Other treatment options include intravitreal injections of triamcinolone acetonide [IVTA] or anti-vascular endothelial growth factor (anti-VEGF) agents [1].

### 1.2. Type 2—Idiopathic Macular Telangiectasia Type 2 or “Perifoveal Telangiectasia”

Idiopathic macular telangiectasia Type 2, also referred to as “perifoveal telangiectasia,” is an acquired condition and represents the most prevalent form of IJFT. Both males and females are equally affected, and the condition typically manifests bilaterally, although it may initially present unilaterally. Yannuzzi et al. reported a mean onset age of 59 years, which is consistent with the mean age of 55 years reported in the Gass–Blodi series [5,6].

The Beaver Dam Eye Study documented a population prevalence of 0.1% (a 95% confidence interval (CI) 0.09, 0.1), whereas The Melbourne Collaborative Cohort Study reported a substantially lower prevalence estimate ranging from 5 to 23 cases per 100,000 individuals. Klein et al. suggested that the variance in prevalence estimates could be attributed to differences in the methodology employed for grading fundus photographs [7,8].

A decline in visual acuity in at least one eye is commonly reported, although functional impairment may be mild initially, with only minimal reduction in binocular best-corrected visual acuity during the early stages [9].

Clemons et al. (2010) found that the mean visual acuity in 522 untreated eyes was 20/40, with 16% of these eyes achieving a visual acuity of 20/20 or better and approximately 50% achieving a vision of 20/32 or better. Similarly, Gass and Blodi reported median visual acuity as 20/40, with 18% of eyes achieving a visual acuity of 20/20 or better [4,10].

Usual alterations in the retina commonly start from the temporal aspect of the paracentral zone and can gradually spread to impact a clearly defined oval area, frequently displaying a longer width horizontally than vertically. When circular involvement occurs, changes typically show more prominence in the temporal area [9].

The main clinical characteristics include small telangiectatic vessels exhibiting fluorescein leakage, a fovea with a cystic appearance lacking intraretinal leakage, and a reduction in retinal transparency [6,9]. Crystalline deposits at the vitreoretinal interface are a characteristic finding that can be observed at different stages of the disease [6]. A diminished foveolar reflex is common and may be noticeable early in the progression of the disease. Nevertheless, a reduced foveolar reflex can also be prevalent among older patients [9]. Observations have included blunted or dilated retinal vessels at right angles, foveal atrophy, subretinal plaques of hyperpigmentation, a round yellow spot, and small retinal hemorrhages [6,9].

Full-thickness or lamellar macular holes have been reported in certain patients as potential complications during the natural course of the disease [9]. Another potential complication is the development of neovascular complexes originating from the retinal vasculature and most frequently located temporally to the foveola. While they initially arise from the retinal vasculature, they may later establish connections to the choroidal vasculature or even lead to choroidal neovascularization [9,11]. Subretinal neovascular structures (SRNVs) develop due to the remodeling and proliferation of retinal capillaries, infiltrating the progressively atrophied outer retina. They are linked to a rapid decline in visual acuity due to exudation, neurosensory detachment, fibrovascular proliferation, and intraretinal and subretinal hemorrhage [1].

All the mentioned clinical characteristics can be thoroughly examined and quantified through a variety of paraclinical investigations, including OCT, OCT-A, fundus autofluorescence (FAF), FA, and adaptive optics imaging (AO) [9].

Gass and Blodi initially divided the natural progression of the disease into five stages [5]. Yannuzzi et al. later simplified it into nonproliferative and proliferative stages, holding therapeutic significance [6].

Several treatment strategies have been explored for MacTel type 2. However, the slow advancement of functional impairments poses challenges in assessing treatment efficacy. Additionally, any potential functional improvements after treatment may be constrained by pre-existing neurosensory atrophy or fibrosis [9].

During the nonproliferative phase of the disease, focal laser photocoagulation and photodynamic therapy (PDT) were found to be ineffective [12]. Extensive research focused on anti-VEGF drugs as a treatment option. However, it remains uncertain whether anti-VEGF injections are beneficial in treating the nonproliferative stage of the disease [1]. Certain publications on intravitreal bevacizumab injections have reported a potential short-term improvement in visual acuity in specific cases [13,14,15,16]. Short-term effects such as a reduction in retinal thickness and angiographic leakage have also been reported following intravitreal bevacizumab injections [13,15].

During the proliferative stage of the disease, the intravitreal administration of anti-VEGF therapy, either alone or in combination with PDT, appeared to be effective [1]. This therapy can be approached due to the fact that anti-VEGF agents have become the established standard of care for most retinal vascular conditions [12].

### 1.3. Type 3—Idiopathic Macular Telangiectasia Type 3 or “Occlusive Telangiectasia”

This is a rare form of IJFT, which is poorly understood due to the scarcity of reported cases. Its primary characteristic is progressive bilateral capillary obliteration, accompanied by telangiectasis and minimal exudation [1]. It is best considered as an ocular manifestation of systemic or cerebral occlusive familial diseases [6].

No cases of occlusive telangiectasia were reported in the Yannuzzi et al. series. Gass and Blodi described seven cases (out of one hundred and forty), among which three had associated systemic vascular occlusive disease, and four had familial ocular cerebral occlusive disorders [5,6].

## 2. Detailed Case Series Description

The present paper outlines a series of three patients diagnosed with MacTel in the Retina Clinic between 2022 and 2023. Each patient underwent a comprehensive ophthalmological assessment, which included visual acuity measurements using ETDRS charts, the slit-lamp examination of both the anterior and posterior segments of the eye, and intraocular pressure measurement. Pupil dilation was induced pharmacologically using 10% Phenylephrine and 1% Tropicamide solutions. Following the initial assessment, retinal imaging was conducted, which included swept-source optical coherence tomography, OCT-A, FAF, and FA, using DRI OCT Triton by Topcon. Subsequently, a series of retinal images were captured using an adaptive optics retinal camera (rtx1TM, Imagine Eyes, Orsay, France). The images encompass the region defined by 1 degree from the fovea, covering all quadrants. To enhance the signal-to-noise ratio, the raw images of the same retinal area were processed using the manufacturer-provided software, resulting in a single final image. The cone mosaic was generated using the manufacturer’s software, i2k Retina AO by Imagine Eyes, France (software version 3.4), which facilitated stitching multiple images obtained from the rtx1 AO retinal camera. All image interpretations were conducted by the same investigator, who had received prior training in retinal image analysis.

**Figure 1:**
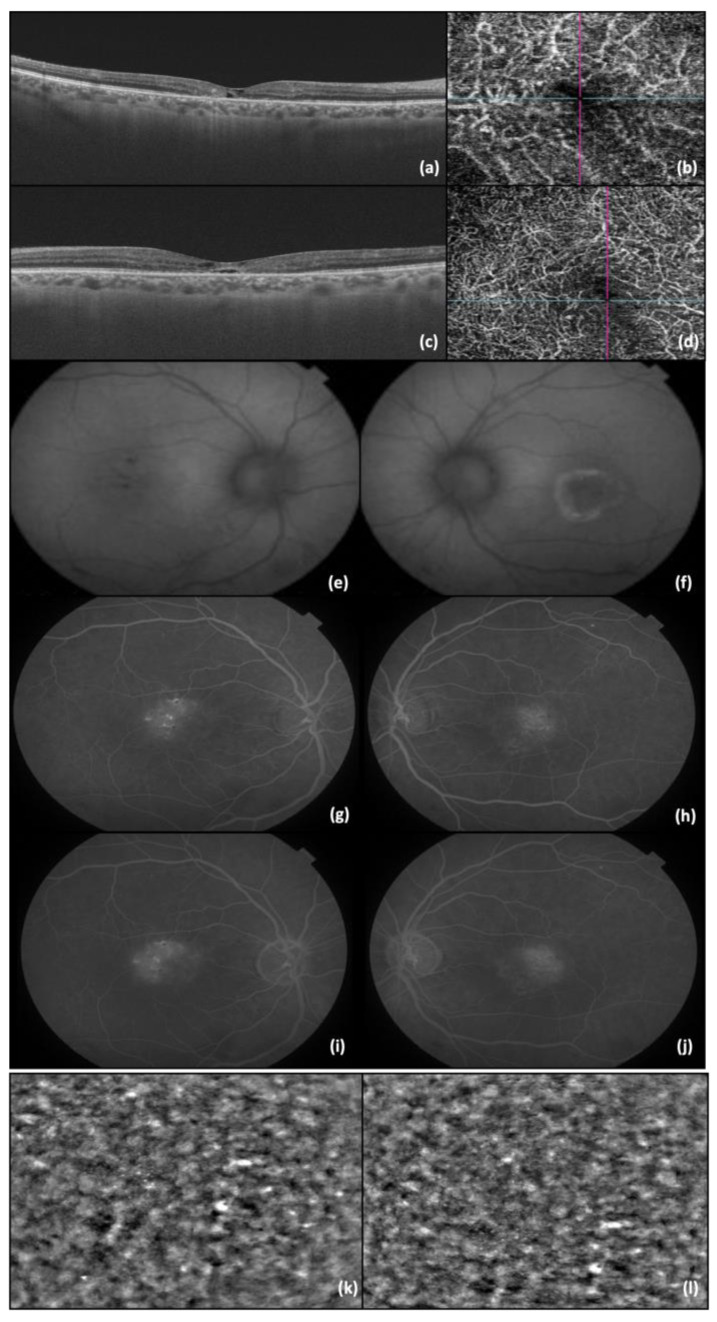
(**a**) OCT image of RE; (**b**) OCT-A image of RE; (**c**) OCT image of LE; (**d**) OCT-A image of LE; (**e**) FAF of RE; (**f**) FAF of LE; (**g**) FA—early phases of RE; (**h**) FA—early phases of LE; (**i**) FA—late phase of RE (**j**) FA—late phase of LE; (**k**) AO image of RE photoreceptors; and (**l**) AO image of LE photoreceptors. The lines in b and d represents the OCT image slice navigators.

The first patient was a 53-year-old man experiencing blurry vision in the last couple of years. BCVA was 0.6 in RE and 0.4 in the LE. An examination of the anterior segment did not exhibit any pathological findings, while the fundus examination revealed a grayish discoloration on the temporal side of the perifovea. The OCT examination showed hyporeflective cavities present in both the inner and outer neurosensory retina, accompanied by outer retinal layer atrophy and the presence of intraretinal hyperreflective foci in the RE. Similarly, in the LE, hyporeflective cavities were observed in the inner neurosensory retina, along with the outer retinal layer atrophy. Using OCT-A imaging, telangiectatic vessels were spotted temporal to the fovea in the deep capillary plexus. FAF revealed decreased autofluorescent spots in the temporal fovea of the RE, while in the LE, a circular perifoveal ring of hyperautofluorescence was observed. Fluorescein angiography detected distinct alterations in BE, identifying the telangiectatic vessels in the early phases, which exhibited diffuse perilesional leakage during the late phases. Adaptive optics images show the cone mosaic pattern (Figure 1).

**Figure 2:**
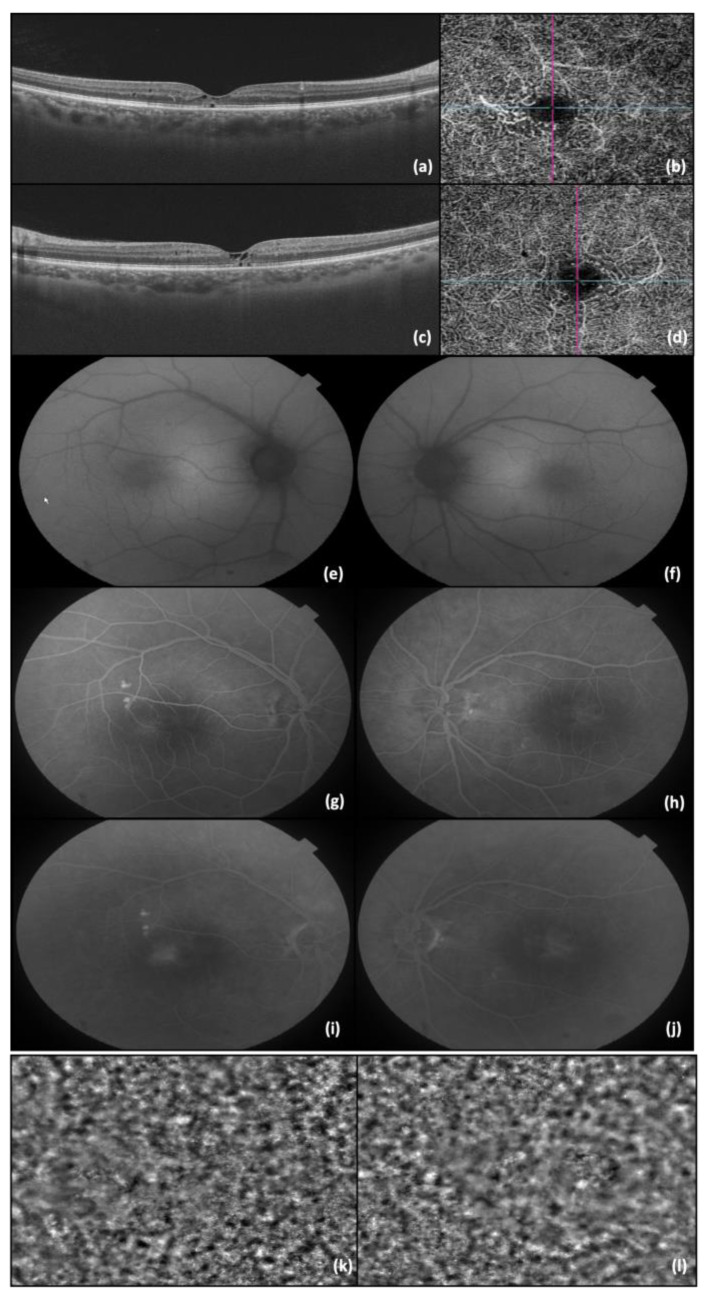
(**a**) OCT image of RE; (**b**) OCT-A image of RE; (**c**) OCT image of LE; (**d**) OCT-A image of LE; (**e**) FAF of RE; (**f**) FAF of LE; (**g**) FA—early phase of RE; (**h**) FA—early phases of LE; (**i**) FA—late phase of RE (**j**) FA—late phase of LE; (**k**) AO image of RE photoreceptors; and (**l**) AO image of LE photoreceptors. The lines in b and d represents the OCT image slice navigators.

The second patient was a 47-year-old man complaining of a gradual decline in visual acuity in both eyes; BCVA was 0.6 in BE when first seen in our clinic. The slit lamp examination was unremarkable for both anterior and posterior poles, except for a slightly diminished foveolar reflex observed in BE. The RE OCT showed hyporeflective cavities in the inner retina, accompanied by disruption of the foveal photoreceptor layer. Similar alterations were observed in the LE, along with the presence of the “ILM drape” sign. In OCT-A imaging, telangiectatic vessels were seen temporal to the fovea in the deep capillary plexus. An FAF examination showed no alterations, whereas the FA examination detected the presence of telangiectatic capillaries in the early phases, followed by diffuse perilesional leakage in later phases. The corresponding cone mosaic pattern was revealed using adaptive optics (Figure 2).

**Figure 3:**
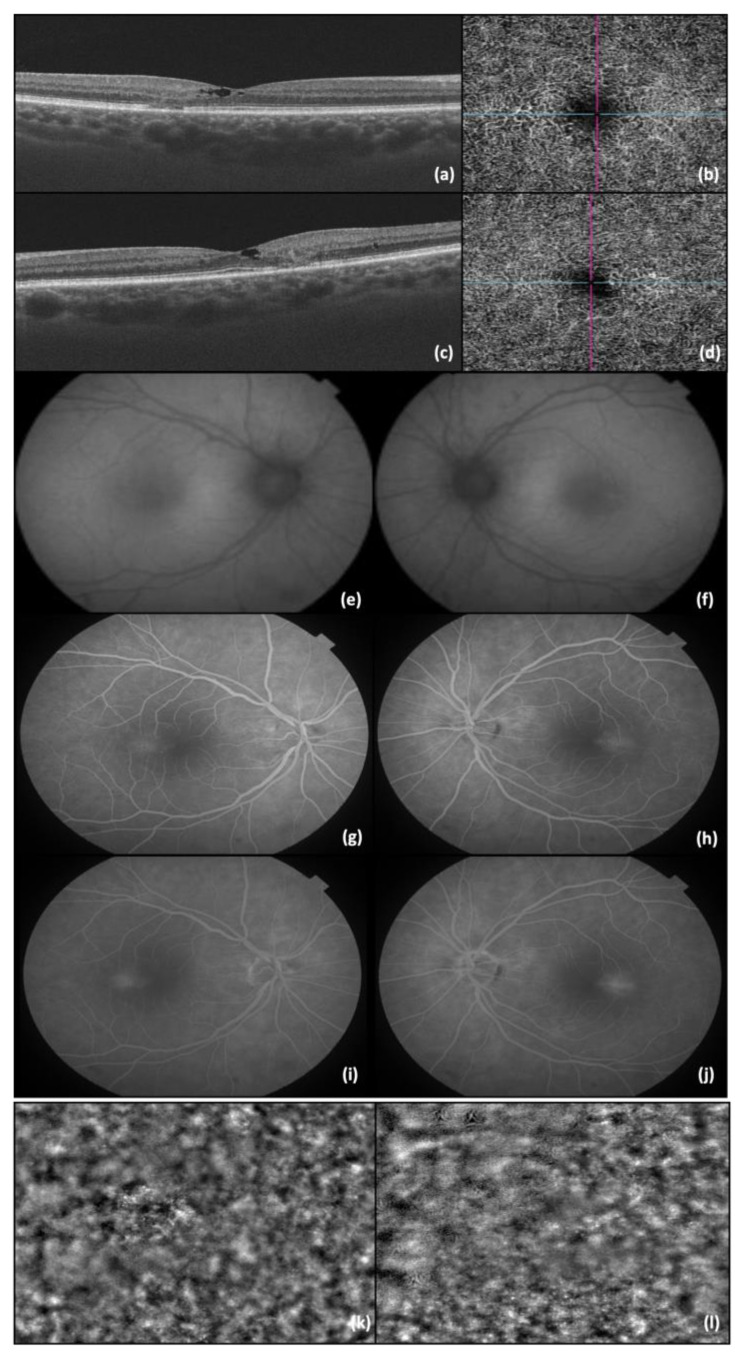
(**a**) OCT image of RE; (**b**) OCT-A image of RE; (**c**) OCT image of LE; (**d**) OCT-A image of LE; (**e**) FAF of RE; (**f**) FAF of LE; (**g**) FA—early phases of RE; (**h**) FA—early phases of LE; (**i**) FA—late phase of RE (**j**) FA—late phase of LE; (**k**) AO image of RE photoreceptors; and (**l**) AO image of LE photoreceptors. The lines in b and d represents the OCT image slice navigators.

The last presented case, and indeed the most challenging one, was a 57-year-old female who visited our clinic complaining of slightly blurred vision in BE (BCVA was 0.9 in BE). The anterior pole examination was within normal limits, while a diminished foveolar reflex was observed in BE during the posterior pole check-up. The OCT showed hyporeflective cavities in the inner retina, accompanied by the loss of the normal outer layer architecture of the foveal region in BE. Using OCT-A imaging, retinal telangiectatic vessels were identified within the deep capillary plexus from the temporal to the fovea. The FAF examination revealed no changes, while the FA examination identified the presence of telangiectatic capillaries, which exhibited diffuse perilesional leakage in the late phases. The appearance of photoreceptors was observed using adaptive optics images (Figure 3).

**Figure 4:**
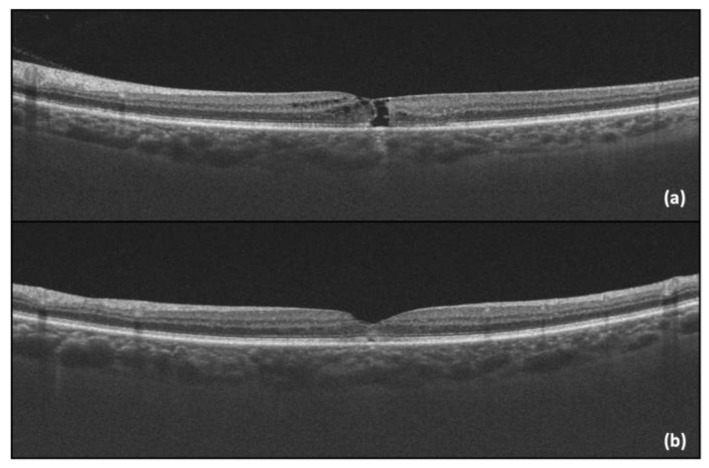
(**a**) LE OCT captured one year after the initial visit, showing the progression into a macular hole with a positive “ILM drape” sign; (**b**) LE OCT captured one month after vitrectomy.

One year after the initial visit, there was a sudden drop in the LE BCVA (LE BCVA = 0.4), corresponding to the progression of the MacTel into a macular hole, with presented an “ILM drape” sign. Pars plana vitrectomy with an inverted flap technique was performed, with subsequent anatomical (OCT capture—Figure 4) and functional improvement (post-operative LE BCVA = 0.6).

**Figure 5:**
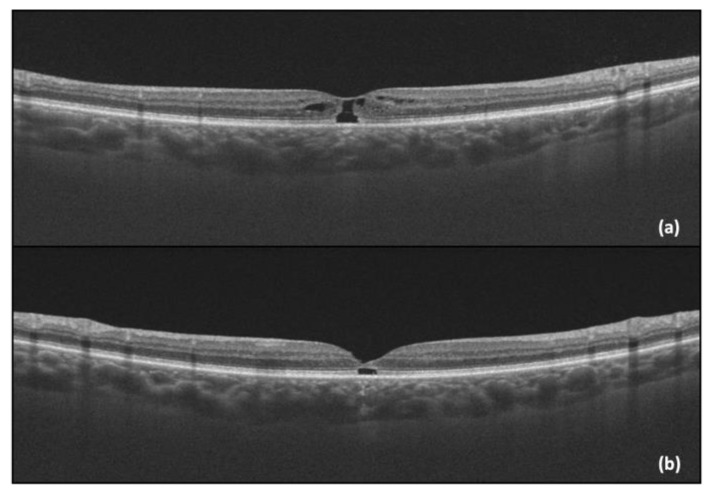
(**a**) RE OCT showing the progression into a macular hole with a positive “ILM drape” sign; (**b**) RE OCT of RE captured one month after vitrectomy.

Two months later, a similar scenario unfolded in the RE, with a BCVA drop from 0.9 to 0.6. The management mirrored that of the left eye, namely pars plana vitrectomy and the inverted flap technique, with favorable postoperative functional (BCVA improvement to 0.8) and anatomical outcomes (Figure 5).

## 3. Discussion

Idiopathic macular telangiectasia Type 2 is an acquired condition and is one of the most common forms of idiopathic juxtafoveal telangiectasia [6]. Its prevalence is low, estimated at 0.1% (95% confidence interval (CI) 0.09, 0.1) according to the Beaver Dam Eye Study, or even lower, ranging from 5 to 23 cases per 100,000 individuals according to The Melbourne Collaborative Cohort Study [7,8]. MacTel is indeed considered to be distributed equally worldwide, with no racial predilection observed. [10]

Diagnostic retinal imaging plays a crucial role in the diagnosis of MacTel type 2 [17]. Optical coherence tomography has also emerged as a valuable tool for both diagnosing and studying MacTel type 2. Early changes in OCT may include temporal enlargement of the foveal pit, resulting in asymmetry with thinning temporally to the fovea. This arises from outer nuclear or Henle’s fiber layer changes. Vascular leakage may obscure this asymmetry by thickening inner retinal layers, causing increased reflectivity. Disruption of the junction between inner and outer photoreceptor segments may be observed, along with early alterations in the photoreceptor outer segment reflectivity [9]. These characteristics may combine with hyporeflective cavities in both the inner and outer neurosensory retina (sometimes the tissue loss creates the “ILM drape” sign, which may be specific to IJFT type 2). Along with these, the outer neurosensory atrophy may also be present in advanced stages. These atrophic spaces may resemble a lamellar or pseudo-lamellar macular hole on funduscopy [1,9]. Another common feature in advanced stages of the disease is the presence of hyperreflective intraretinal or subretinal lesions, which are attributed to pigment migration or neovascular membranes [9].

Macular teleangiectasia is an ideal candidate for optical coherence tomography angiography imaging. Early in the course of this disease, there are retinal microvasculature changes, primarily impacting the temporal side of the parafoveal deep capillary plexus. As the disease advances, microvascular abnormalities extend around the fovea and into the superficial capillary plexus. This leads to the formation of dilated anastomoses between these plexuses, resulting in retinal atrophy, cysts, and photoreceptor outer segment loss. In certain instances, these anastomoses can evolve into subretinal neovascularization, linking with the choroidal vasculature [18].

The OCT and OCT-A characteristics mentioned above have been observed in our patients, supporting our diagnostic assessment.

Fundus autofluorescence is a crucial non-invasive tool for diagnosing and monitoring macular telangiectasia. FAF imaging reveals distinct patterns that correlate with disease progression and retinal structural changes. Wong et al. identified four categories of FAF patterns corresponding to disease severity: category 0 (normal eyes) showed normal FAF, category 1 (early disease) displayed mild increased foveal autofluorescence, category 2 (mild to moderate disease) demonstrated a further increase in FAF with clinical MacTel features, category 3 (advanced disease) exhibited marked FAF increases and foveal atrophy and category 4 (severe disease with pigment clumping) showed mixed FAF patterns with advanced disease and pigment clumping [19]. Increased fundus autofluorescence in the fovea is thought to precede clinical and angiographic findings in macular telangiectasia. This likely results from macular pigment depletion rather than increased lipofuscin accumulation in the retinal pigment epithelium or pigment displacement. The distribution of macular pigment density correlates with the extent of leakage observed on fluorescein angiography. Initially, there is a decrease in macular pigment density, which is likely attributed to the loss of xanthophyll pigments, followed by a temporal spread to the entire fovea and the development of a circular perifoveal ring. While disruptions in the macular pigment can occur with other ocular diseases, this pattern is unique to macular telangiectasia [20,21].

Fluorescein angiography is a key diagnostic tool for macular telangiectasia, often detecting leakage before visible changes. Early FA phases show dilated leaking telangiectatic capillaries temporal to the fovea, followed by diffuse hyperfluorescence in the later phases. Notably, leakage in macular telangiectasia does not cause retinal edema or thickening, suggesting a partial breakdown of the blood–retinal barrier and allowing for the passage of fluorescein but not albumin [20,21].

Similar findings have been observed in our patients on both FAF and FA.

Adaptive optics is a technique used to correct optical wavefront aberrations of the human eye, which is not optically perfect due to the presence of these aberrations. These imperfections not only impact visual discrimination and recognition but also restrict the observation of delicate ocular structures, impeding our understanding of eye disease mechanisms. When incorporated into ophthalmoscopes, AO allows for retinal imaging at the cellular level [22]. Integrating adaptive optics with technologies like a scanning laser ophthalmoscope enables the in vivo examination of the photoreceptor cone mosaic [9]. Using this technique, Ooto et al. discovered a reduced paracentral cone density in patients with macular telangiectasia type 2 compared to normal individuals. Additionally, certain paracentral regions exhibited cone loss, even in areas where no leakage was observed on fluorescein angiography [23]. Adaptive optics provides detailed insight into diseases like macular telangiectasia. Given this finding, it is essential to further investigate the concept that foveal cone photoreceptor loss may precede or coincide with vascular changes in this condition [24]. Our patients also benefited from adaptive optics investigation.

Long-term prognosis in macular telangiectasia type 2 varies, with most patients maintaining VA better than 0.2. Vision loss starts paracentrally, progressing centrally over time. Severe central vision loss and legal blindness can occur with proliferative disease [17]. In the case series presented in the current paper, BCVA at presentation ranged between 0.4 and 0.9, with all patients demonstrating a nonproliferative stage of the disease. The only significant visual drop was noted when there was progression to a macular hole. Vitrectomy with the inverted flap technique was the surgical approach of choice due to its favorable postoperative prognosis.

## 4. Conclusions

Macular telangiectasia type 2 is a rare retinal disease characterized by abnormalities in the macular blood vessels. Its prevalence is estimated to be around 0.1%, affecting both genders equally.

Investigations for this condition include a comprehensive ophthalmic evaluation, fundus imaging, OCT, OCT-A, FA, and FAF. Additionally, advanced imaging modalities, such as adaptive optics, provide detailed visualization of the retinal microstructure, revealing the intricate cone mosaic pattern of the retina. These techniques enhance our understanding of MacTel type 2 and aid in monitoring disease progression and treatment efficacy.

While the prognosis varies, most patients maintain visual acuity better than 0.2, although progression can lead to severe central vision loss and legal blindness in some cases.

The management of MacTel type 2 involves tailored approaches for both nonproliferative and proliferative forms of the disease. In the nonproliferative stage, close monitoring of visual acuity and disease progression is essential. For proliferative disease, interventions like anti-VEGF therapy or photocoagulation may be employed to manage complications such as subretinal neovascularization. Additionally, true lamellar or full macular holes, which are potential complications of MacTel type 2, may require surgical intervention such as vitrectomy with membrane peeling to restore visual function.

Regular follow-up is crucial for optimizing outcomes and addressing complications effectively in MacTel type 2.

## Data Availability

The original contributions presented in the study are included in the article; further inquiries can be directed to the corresponding author/s.
